# Gene Expression Profile and Functionality of ESC-Derived Lin-ckit+Sca-1+ Cells Are Distinct from Lin-ckit+Sca-1+ Cells Isolated from Fetal Liver or Bone Marrow

**DOI:** 10.1371/journal.pone.0051944

**Published:** 2012-12-27

**Authors:** Irina Fernandez, Krista M. Fridley, Dhivya Arasappan, Rosalind V. Ambler, Philip W. Tucker, Krishnendu Roy

**Affiliations:** 1 Department of Biomedical Engineering, The University of Texas at Austin, Austin, Texas, United States of America; 2 Department of Molecular Genetics and Microbiology, The University of Texas at Austin, Austin, Texas, United States of America; 3 The Institute for Cell and Molecular Biology, The University of Texas at Austin, Austin, Texas, United States of America; 4 Genome Sequencing and Analysis Facility, The University of Texas at Austin, Austin, Texas, United States of America; French Blood Institute, France

## Abstract

*In vitro* bioreactor-based cultures are being extensively investigated for large-scale production of differentiated cells from embryonic stem cells (ESCs). However, it is unclear whether *in vitro* ESC-derived progenitors have similar gene expression profiles and functionalities as their *in vivo* counterparts. This is crucial in establishing the validity of ESC-derived cells as replacements for adult-isolated cells for clinical therapies. In this study, we compared the gene expression profiles of Lin-ckit+Sca-1+ (LKS) cells generated *in vitro* from mouse ESCs using either static or bioreactor-based cultures, with that of native LKS cells isolated from mouse fetal liver (FL) or bone marrow (BM). We found that *in vitro*-generated LKS cells were more similar to FL- than to BM LKS cells in gene expression. Further, when compared to cells derived from bioreactor cultures, static culture-derived LKS cells showed fewer differentially expressed genes relative to both *in vivo* LKS populations. Overall, the expression of hematopoietic genes was lower in ESC-derived LKS cells compared to cells from BM and FL, while the levels of non-hematopoietic genes were up-regulated. In order to determine if these molecular profiles correlated with functionality, we evaluated ESC-derived LKS cells for *in vitro* hematopoietic-differentiation and colony formation (CFU assay). Although static culture-generated cells failed to form any colonies, they did differentiate into CD11c+ and B220+ cells indicating some hematopoietic potential. In contrast, bioreactor-derived LKS cells, when differentiated under the same conditions failed to produce any B220+ or CD11c+ cells and did not form colonies, indicating that these cells are not hematopoietic progenitors. We conclude that *in vitro* culture conditions significantly affect the transcriptome and functionality of ESC-derived LKS cells and although *in vitro* differentiated LKS cells were lineage negative and expressed both ckit and Sca-1, these cells, especially those obtained from dynamic cultures, are significantly different from native cells of the same phenotype.

## Introduction

Hematopoiesis is a complex and highly ordered process in which hematopoietic stem cells (HSCs) give rise to mature blood cells. During later stages of embryogenesis and until birth, hematopoiesis occurs in the fetal liver [1]. However, during adulthood the maintenance and differentiation of HSCs occurs in the bone marrow. In mice, most long-term multi-lineage HSC activity resides within the lineage-negative, ckit-positive, and Sca-1 positive fraction (Lin-ckit+Sca-1+, LKS cells) of murine bone marrow and fetal liver [2]–[4]. These cells have been shown to reconstitute *in vivo* all hematopoietic cells in mice following irradiation and hemato-lymphoid lineage depletion [5]–[7]. Similarly, the human CD34+,Thy-1+,CD38−,CD45RA-cell population contain HSCs capable of giving rise to hematopoiesis after transplantation into xenogeneic models of myeloablated immunodeficient mice [8]. The clinical relevance of these cells has been further confirmed in humans after autologous HSC-rescue blood formation in myeloablated recipients, providing sustained hematopoiesis [8]. Still, usage of donor HSCs in clinical practice suffers significant limitations, including limited availability of human leukocyte antigen (HLA)-matched donors, morbidity associated with bone marrow collection as well as isolation, and propagation of freshly isolated HSCs *ex vivo*. Therefore, HSCs derived *in vitro* from embryonic or induced-pluripotent stem cells (ES or iPS cells) could provide an on-demand, readily available cell source for a variety of therapeutic applications. However, the clinical applicability of these ES/iPS-derived cells depends critically on (a) efficient methods for differentiation and expansion and (b) whether these cells are genetically and functionally equivalent to their native, *in vivo* counterparts from bone marrow (BM) or fetal liver (FL).

We and others have recently shown, that LKS cells can be efficiently generated *in vitro* from both embryonic and induced pluripotent stem cells (ESCs and iPSCs) ([9]–[11] reviewed in [12]). During differentiation in suspension cultures, ESCs form aggregates known as embryoid bodies (EBs). Similar to embryonic development, EBs increase in size and complexity in culture and differentiate into the three germ layers of embryonic development: endoderm, ectoderm, and mesoderm. Subsequently, the mesoderm gives rise to blood tissue and lineage-specific cells including HSCs. Mouse ESCs have been differentiated *in vitro* by using traditional two dimensional (2D), static culture systems and, more recently, by using several different types of bioreactors, including spinner flasks and rotary wall vessels [13]. Unlike traditional static culture methods, bioreactor systems have the ability to achieve scale-up and be integrated with chemical process development–two parameters critical for potential clinical applications. Our laboratory has recently demonstrated efficient generation of LKS cells in both stirred (spinner flask) and rotary-wall bioreactors and evaluated the global gene expression profile of ESCs differentiated in these bioreactor systems [14], [15]. However, despite significant progress in generating ESC-derived LKS cells, a cell fraction which has been presumed to be enriched in HSCs, very few studies have evaluated whether these *in vitro* generated cells are genetically and functionally equivalent to *in vivo* LKS populations derived from BM or FL. Such detailed comparison of ESC- or iPSC-derived LKS cells with corresponding native phenotypic counterparts is critical for examining their ability to function as replacements for donor-derived stem cells in clinical therapies. It is also important to understand how various culture conditions affect the gene expression profile of *in vitro* derived progenitor-like cells expressing a common set of surface markers.

Isolated mouse cell populations enriched in stem cells have been previously studied by other groups [16]–[22] to compare the gene expression profiles of native LKS cells from different tissues or to decipher genes specifically expressed in embryonic stem cells during pluripotency and differentiation (reviewed in [23]). In this study, we compared the gene expression profiles of LKS cells isolated from *in vitro* differentiated mouse ESCs under various static and dynamic bioreactor culture conditions with native LKS cells from mouse BM or FL in order to determine the ability of ESC-derived LKS progenitors to function as replacements for native adult stem cells in clinical therapies. We show that although all these cells expressed both ckit and Sca-1 on their surface, ESC-derived LKS cells from bioreactors have impaired hematopoietic potential and differ both functionally and in terms of their transcriptome, from LKS cells generated in static culture as well as from LKS cells isolated from *in vivo* hematopoietic niches.

## Methods

All animal work was conducted in accordance to protocols approved by the Institutional Animal Care and Use Committee (IACUC) of The University of Texas at Austin.

### ESC Expansion and Differentiation via Embryoid Body (EB) Formation

Mouse R1 ESCs [24] were obtained from Dr. A. Nagy (Mount Sinai Hospital, Ontario, Canada). ESCs were expanded on inactivated-mouse embryonic fibroblasts (MEF cells, ATCC, Manassas, VA, USA). ESCs were differentiated in suspension cultures either in Static flasks (Ultra Low Attachment Flask, Corning Incorporated, Corning, NY,USA), a Spinner flask system (125 ml Disposable Spinner Flask, Corning, NY,USA), or Synthecon rotating vessels (22.5 ml Slow Turning Lateral Vessel, Rotary Cell Culture System, Synthecon Inc., Houston, TX,USA) using previously optimized cell seeding densities and rotation speeds that produced the highest percentage of ckit^+^Sca-1^+^ cells for each culture system (500,000 cells/ml for Static and Synthecon cultures and 750,000 cells/ml for spinner flasks with rotation speed of 20 rpm for Synthecon vessels and 100 rpm for spinner flasks) [14]. Single-cell suspensions were obtained from day 7 EBs of each condition as previously described [14].

### Isolation of Mouse Bone Marrow and Fetal Liver Cells

129X1/SVJ and 129S1/SvImj mice (Jackson Lab, USA) were bred to obtain identical MHC background to R1 ESCs. Mice were maintained at University of Texas at Austin Animal Research Center (ARC). Whole bone marrow (BM) was isolated from femurs and tibias of 8–12 week-old mice, and fetal livers (FL) were harvested from E15 timed pregnancies of 129X1/SVJ X 129S1/SvImj matings. Single-cell suspensions were prepared as described [25] with minor modifications.

### Fluorescence-activated Cell Sorting of Lin-ckit+Sca-1+ Cells

BM and FL cells were incubated with a cocktail of biotinylated antibodies against a panel of lineage hematopoietic antigens (CD5, CD45R (B220), CD11b, Gr-1 (Ly-6G/C), 7–4, and Ter-119) following the manufacturer’s protocol (Lineage cell Depletion kit 130-090-858, Miltenyi Biotech, Germany). For day7 EBs, the Lineage negative cocktail was not included for sorting because lineage positive cells were not observed at day 7 of differentiation for any culture condition. Single-cell suspensions from BM and FL lineage negative cells, as well as from day 7 EBs, were stained with anti-mouse CD16/CD32 Fc Block for 10 min at 4°C followed by ckit-APC and Sca-1-PE antibodies (Cat No. 553108 and 553356, BD Biosciences,USA) for 30 minutes at 4°C. Staining with rat IgG2a-PE and rat IgG2b-APC isotype controls were included. Live cells were distinguished from dead cells by gating using the forward and side scatter signals as well as by exclusion of 7-amino-actinomycin D (eBioscience, USA) stained dead cells. Cell sorting was performed on an FACSAria Cell sorter (BD Biosciences,USA). Analyses of the Lin-ckit+Sca-1+ sorted cells were performed to determine sorting purity and to evaluate the Mean Fluorescence Intensity (MFI) of ckit and Sca-1 expression on sorted cells from each condition. Following sorting, Lin^-^ckit^+^Sca-1^+^ cells from each sample were resuspended in lysis buffer (RLT, Qiagen, Germany). RNA was isolated according to manufacturer’s protocol (RNeasy mini kit, Qiagen, Germany).

### Microarray Processing and Analysis

Samples for mRNA profiling studies were processed by Asuragen, Inc, USA, according to the company’s standard operating procedures. The purity and quantity of total RNA samples were determined by absorbance readings at 260 and 280 nm using a NanoDrop ND-1000 UV spectrophotometer. The integrity of total RNA was qualified by Agilent Bioanalyzer 2100 capillary electrophoresis. Total RNA (100 ng per sample) was used for preparation of biotin-labeled targets (cRNA) using a MessageAmp™ II-based protocol (Ambion Inc., Austin, TX,USA) and one round of amplification. The cRNA yields were quantified by UV spectrophotometry and the distribution of transcript sizes was assessed using the Agilent Bioanalyzer 2100 capillary electrophoresis system. Labeled cRNA was used to probe MouseRef-8 v2 Expression BeadChips (BD-202-0202,Illumina). The Mouse Ref-8 V2 expression BeadChip targets approximately 25,600 well-annotated RefSeq transcripts encoded from over 19,100 unique genes. Hybridization, washing, and scanning of the Illumina arrays were carried out according to the manufacturer’s instructions. Briefly, cRNA/hybridization buffer mixtures were incubated at 65°C for 5 minutes, followed by brief centrifugation to collect. The mixtures were added to BeadChips and then BeadChips inserted into hybridization chambers, and hybridization was carried out at 58°C for 16–18 hours at rocking speed set to 5. BeadChips were washed and stained in the following steps: 1) 55°C for 10 minutes static in HighTemp Wash Buffer in a Hybex waterbath, 2) ambient temperature shaking for 5 minutes in E1BC wash buffer, 3) ambient temperature shaking for 10 minutes in ethanol, 4) ambient temperature shaking for 2 minutes in E1BC buffer, 5) ambient temperature for 10 minutes rocking in Block E1 buffer, 6) ambient temperature for 10 minutes rocking in Block E1 buffer containing 1 ug/ml Cy3-streptavidin, and 7) ambient temperature shaking for 5 minutes in E1BC buffer. BeadArrays were dried in a centrifuge for 4 minutes and scanned using an Illumina BeadArray Reader. Scanner settings were set at a default, Factor 1 setting, and scanned images were visually examined for the presence of anomalies and to ensure that average P95 values for each array is greater than 500. Illumina BeadScan software was used to produce idat, .xml, and.tif files for each array on a slide and.sdf files for each barcode on a slide of 8 arrays.

Raw data were extracted using Illumina BeadStudio software (v 3). Following quality assessment, data from the replicate beads on each array were summarized into raw intensity values with and without background subtraction in an Excel report containing the project description (sample key), gene identifiers and corresponding probe IDs, table of detection *p*-values, tables of normalized data with and without background subtraction, array quality control metrics, and array quality control metric cut-offs and definitions. The following array quality control metrics were met: hybridization control graphs must have increasing signal intensities, negative graph must show an average background of less than 200, and the average biotin signal must be 6000 or higher.

### Normalization and Statistical Analysis of Microarray Data

For microarray data, background subtraction, expression summary, normalization, and log base 2 transformation of gene signals were carried out using Quantile Normalization [26]. For statistical analysis of gene expression, one-way ANOVA was used for multiple group comparisons across all samples in the experiment, followed by multiple testing correction to determine the false discovery rate (FDR) [27]. Genes with a FDR-adjusted *p-*value of <0.05 were considered differentially expressed genes (DEG). Pair-wise comparisons were then performed for all DEG. For each pair of treatments, a two-sample *t*-test was carried out for every gene, followed by a multiple testing correction to determine FDR. Genes with a FDR-adjusted *p-*value of <0.05 were considered statistically significant. The mean of the intensity value for each gene (three biological replicates from each group) was calculated and graphically represented by hierarchical clustering selecting all 13,000+ DEG across all conditions. The BM and FL samples were grouped together (BM+FL) as were the Spinner and Synthecon (Spinner+Synthecon) samples. Log ratios of Static vs BM+FL group and Spinner+Synthecon group vs BM+FL group were calculated. Any gene showing 5-fold difference in these comparisons was selected. Hierarchical clustering was done and a heat map was generated using MeV 4.8.1. Separate heat maps were also generated using just the genes that were up regulated in comparison to the BM+FL group, and using just the genes that were down regulated in comparison to the BM+FL group. Differentially expressed genes (p<0.05) whose expression differed 5-fold from BM and FL-LKS cells to Lin-ckit^+^Sca-1^+^ cells generated in Static, Spinner flask, and Synthecon rotary-wall cultures were compared using a Venn diagram [28] to identify similar and unique genes between groups and analyzed using Gene Ontology (GO) Analysis for functional annotation [29].

Statistical analyses of flow cytometry data (MFI analysis) were performed using Systat (Systat Software, Inc.) with an ANOVA and a significance level of p<0.05. The equality of variances was determined using a Levene’s test followed by Tukey’s posthoc analysis for samples with equal variance and a Games-Howell posthoc analysis for samples with unequal variances, as determined appropriate by the Levene’s test.

### Hematopoietic Differentiation of ESC-derived and BM LKS Cells

Static and Dynamic (Synthecon)-culture derived Lin-ckit+Sca-1+ sorted cells, as well as Lin-ckit+Sca-1+ sorted bone marrow cells were cultured in 24-well plates (0.5×10^6^ cells/well) in RPMI-1640 medium supplemented with 10% FCS in the presence of recombinant murine GM-CSF (20 ng/ml), IL-4 (10 ng/ml) and IL-3 (10 ng/ml), all from Peprotech,USA. Half of the culture medium was changed on day 3 and day 5. On day 6, non-attaching cells were harvested by pipetting and stained for Flow cytometry to evaluate expression of hematopoietic cell markers: CD11c, clone N418 (anti-CD11c-FITC Cat.No.11-0114-81) and B220, clone RA3-6B2 (anti-B220-FITC Cat.No.11-0452-81), both from eBiosciences, CA, USA).

### In vitro Colony Forming Unit (CFU) Assay

Methylcellulose-based medium (Methocult, CatNo.GF M3434, Stem Cell Technologies, BC, Canada) was used to detect and quantify hematopoietic progenitor cells in a Colony-forming Unit (CFU) assay. *In vitro*-derived Lin-ckit+Sca-1+ sorted mouse ESCs (Static and Synthecon), as well as Lin-ckit+Sca-1+ mouse BM cells were assessed following manufacturer’s protocol. Briefly, 100, 1000 and 10000 sorted cells/35 mm dish containing 1.1 ml of the cultured medium was plated. For *in vitro*-derived Lin-ckit+Sca-1+ mouse ESCs, an extra plate of 100,000 cells/dish was included. The mixture was incubated under a humidified incubator (5% CO2 at 37°C). Colonies were visualized after 8–12 days of culture.

## Results

### ESC Differentiation into Lin-ckit+Sca-1+ Cells Under Static and Dynamic Culture Conditions

**Figure 1:**
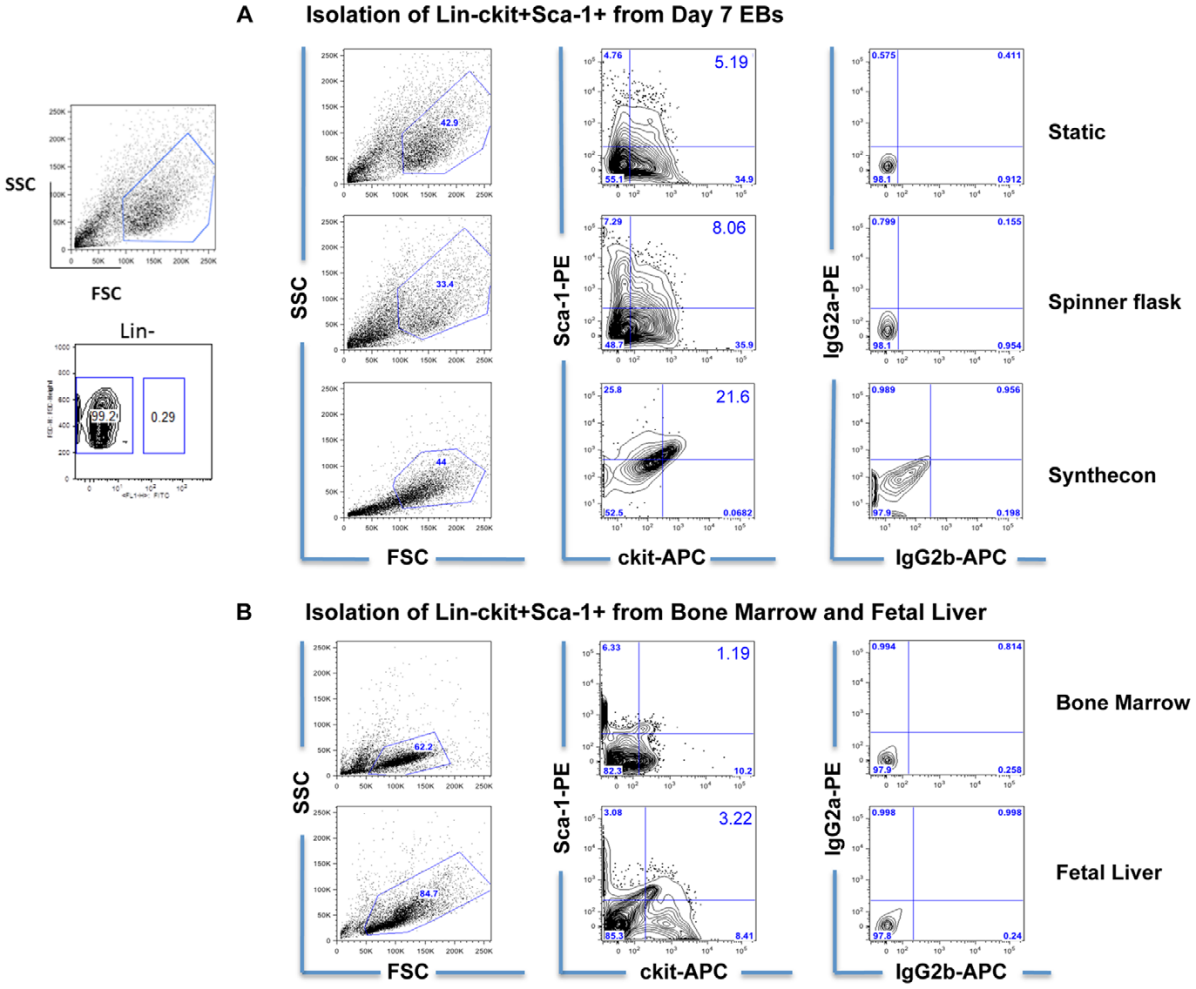
Identification and isolation of ES cells and BM- and FL-derived Lin-ckit+Sca-1+ cells. (**A**) Lin-ckit+Sca-1+ cells were generated from ES cells differentiated under Static and Dynamic culture conditions (Synthecon and Spinner flask) for 7 days and (**B**) from BM and FL. Mouse embryonic stem cells were differentiated under Static and Dynamic culture conditions (Spinner flask and Synthecon) for 7 days. Cell suspensions from day7 EBs were stained with ckit and Sca-1 antibodies to isolate the Lin-ckit+Sca-1+ population. As explained in Materials and Methods, the Lineage negative cocktail was not included for sorting because we did not observe lineage positive cells at day 7 of differentiation for any ESC culture condition (left panel). Similarly, lineage negative cells from BM and FL cell suspensions were stained with ckit and Sca-1 antibodies to isolate by cell sorting the ckit+Sca-1+ Lineage negative population (LKS). Staining with relevant isotype controls was included in each sample to determine the right ckit+Sca-1+ population.

Mouse ESCs were spontaneously differentiated using static and dynamic (Spinner flask and Synthecon) suspension cultures. We recently demonstrated that cell seeding density and bioreactor speed in both Spinner Flask and Synthecon systems significantly alters efficiency of ESCs differentiation into Lin-ckit+ Sca-1+ cells [14]. However, it is unclear how different *in vitro* culture conditions affect the gene expression profiles of ESC-derived cells that express a common set of surface markers in contrast to more customary assays of the entire heterogeneous population of differentiating cells. In order to evaluate that, we isolated the Lin-ckit+Sca-1+ population from each culture condition (day 7 EBs) by flow cytometry-based cell sorting. As shown in **Figure 1A**, there were no detectable lineage positive cells (Lin+ committed hematopoietic cells) in any culture condition at day 7 of differentiation, indicating efficient isolation of the Lin-ckit+Sca-1+ cell population. Consistent with our previous results [14], a higher percentage of Lin-ckit+Sca-1+ cells were observed under dynamic conditions (Spinner and Synthecon) as compared to Static cultures. Similarly, lineage negative (Lin-) ckit+Sca-1+ cells were isolated from mouse BM and FL cells. Detailed surface marker staining for these BM and FL-derived cells are shown in **Figure 1B**, which indicates efficient isolation of the LKS population. Additionally, the purity of Lin-ckit+Sca-1+ sorted cells was confirmed (**Figure S1a**). Interestingly, mean fluorescence intensity (MFI) analyses demonstrated that bioreactor (both Synthecon and Spinner)-derived cells had increased Sca-1 expression compared to FL, BM and Static-derived cells (**Figure S1b**). In addition, BM cells had increased MFI of ckit compared to all other ESC-derived conditions. This differential MFI on the sorted populations translates into differential expression levels of Sca-1 and ckit across the different conditions, possibly explaining their different gene expression profiles and degrees of hematopoietic commitment.

### Global Gene Expression of ESC-, BM-, and FL-derived Lin^-^ckit^+^Sca-1^+^ Cells

**Figure 2:**
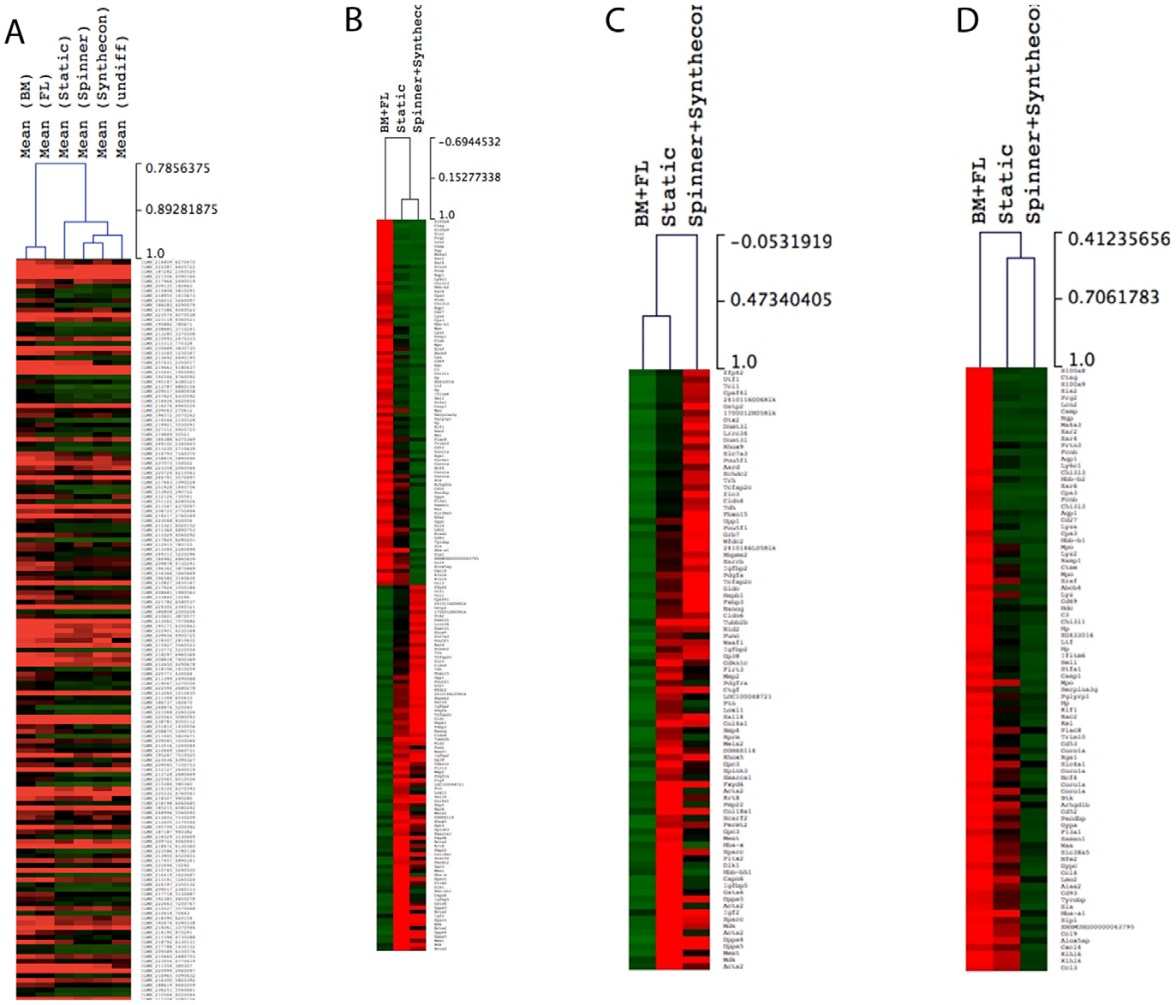
Global gene expression profile of BM- and FL-isolated as well as in vitro generated Lin-ckit+Sca-1+ progenitor cells. **A)** Sample Hierarchical clustering represents the conditions that cluster together based on differentially expressed genes (DEG) across all samples (mean of gene expression values for each group, One way Anova, p<0.05). **B)** Gene Hierarchical clustering of 5-fold DEG across native (BM+FL),Static and Dynamic (Spinner+Synthecon) culture conditions. Gene Hierarchical clustering of 5-fold DEG up-regulated (**C**) or down-regulated (**D**) in Static and Dynamic (Spinner and Synthecon) *in vitro* culture conditions compared to native isolated LKS cells (BM+FL). The genes used in these heat maps are listed within Supplementary material.

We compared mouse ESC-derived LKS cells, generated either in Static suspension cultures, dynamic Synthecon™ rotary-wall vessels or Spinner flasks, to LKS cells isolated from BM and FL. We focused our analysis on genes for which significant differences in expression were identified between groups (Static, Spinner flask, Synthecon, BM, and FL). The global gene expression profile of each group, including mouse ESCs at day 0 of differentiation (undifferentiated cells) is represented in a hierarchical clustering in **Figure 2A**. 13497 genes were differentially expressed across all samples. Overall, and as expected, the global gene expression pattern was similar across all samples when all DEG were included. However, there are differences that resulted in sample clustering. As shown in **Figure 2A**, the gene expression pattern of each gene when compared between BM and FL was highly similar, grouping them together. Interestingly, BM and FL-derived LKS cells were more similar to Static culture-generated LKS cells and more different from both Dynamic (Spinner and Synthecon) culture-derived LKS cells (**Figure 2A and 2B**). Overall, all ESC-derived LKS groups were more similar to undifferentiated ESCs compared to BM and FL-derived LKS cells. However, undifferentiated cells cluster together with Spinner and Synthecon and separately from Static cultures. These results suggest that the LKS population from Static cultures at day 7 is enriched in cells more committed to hematopoietic lineage compared to their Dynamic counterparts.

To determine the differences in gene expression profile between Static and Dynamic-derived LKS cells compared to native LKS cells, the BM and FL samples were grouped together (BM+FL) as were the Spinner and Synthecon samples (Spinner+Synthecon). Any gene showing 5-fold difference in these comparisons was selected. Hierarchical clustering was performed, and a heat map was generated (**Figure 2B**). Separate heat maps were also generated using just the genes that were up-regulated in comparison to the BM+FL group (**Figure 2C**), and using just the genes that were down-regulated in comparison to the BM+FL group (**Figure 2D**). Interestingly, Static-derived and native LKS cells (BM+FL) cluster together when the up-regulated genes were selected (**Figure 2C**). In contrast, Static and Dynamic cultures (Spinner and Synthecon) cluster together when genes that are down-regulated are selected (**Figure 2D**). The list of genes used in these heat maps is provided as part of Supplementary material (**Tables S5 and S6**).

**Figure 3:**
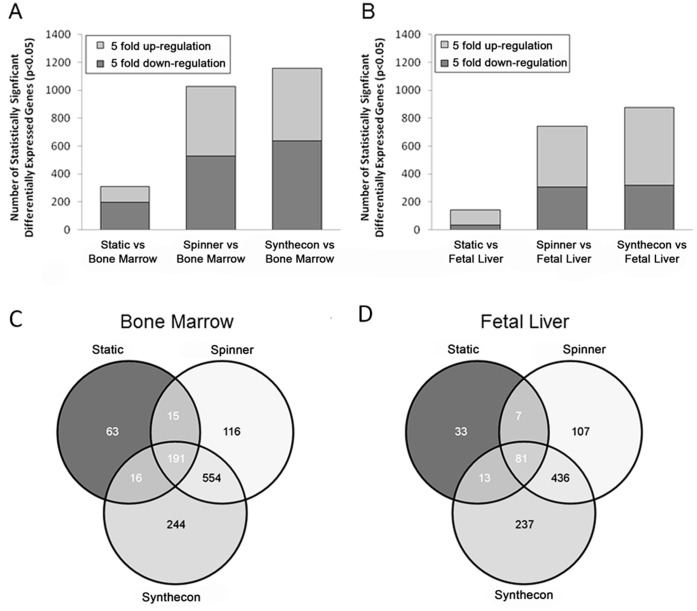
Differentially expressed genes in various LKS cell populations. Numbers of differentially expressed genes in Lin-ckit+Sca-1+ progenitor cells generated under Static, Spinner flask, and Synthecon rotary culture conditions with a 5-fold difference compared to LKS cells from bone marrow and fetal liver. ESC-derived Lin-ckit+Sca-1+ cells from all ESC conditions had fewer differentially expressed genes (p<0.05) relative to FL LKS cells (**B**) than to BM LKS cells (**A**). Expression profiles derived from Static culture conditions were more similar to BM and FL LKS cells expression patterns than were those generated under dynamic (Spinner flask or Synthecon) conditions. Venn diagram of genes differentially expressed (p<0.05) in Lin-ckit+Sca-1+ progenitor cells generated under Static, Spinner flask, and Synthecon rotary culture conditions with a 5-fold difference compared to LKS cells isolated from bone marrow (**C**) or fetal liver (**D**). Many genes were differentially expressed only within a particular culture system, and the dynamic culture systems shared the largest number of differentially expressed genes compared to both BM (**C**) and FL (**D**).

As observed in **Figure 3A and B**, significantly differentially expressed genes from all LKS cells generated *in vitro* were more similar to FL- than BM-LKS cells. Among all *in vitro* LKS cells, Static culture generated gene expression profiles most similar to native LKS cells. Expression profiles for each of the three ESC conditions were more similar to each other than to BM or FL derived LKS cells (data not shown). The relationship between LKS cells generated in static, Spinner flask, and Synthecon cultures, were plotted as a Venn diagram to show the intersection and differences of differentially expressed genes compared to either BM or FL (**Figure 3C and D**). The larger number of intersecting genes in the Spinner flask and Synthecon groups demonstrated that Dynamic cultures were more similar to each other and differed from Static cultures.

Identification of Gene Ontology (GO) groups focused on genes with the lowest p-values that were derived from unique term lineages. In general, genes up-regulated in ESC cultures compared to BM and FL, included GO terms involved in development (**Table S1 and S3**). Down-regulated genes in ESC conditions, when compared to BM and FL, contained GO terms important for hematopoietic cell functionalities (**Table S2 and S4**). These results imply that ESC-derived LKS cells are still largely in a developmental stage, rather than a stage expressing genes characteristic of multipotent hematopoietic cells.

### Hematopoietic Gene Expression in ESC Cultures Compared to Bone Marrow and Fetal Liver

**Figure 4:**
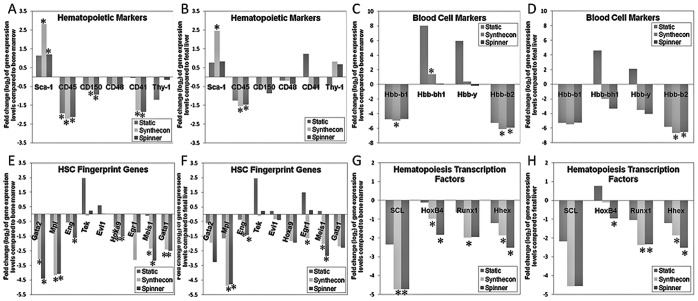
Differences in LKS cell gene expression. Fold changes of lineage-specific genes differentially regulated in Lin-ckit+Sca-1+ cells derived from Static and bioreactor culture conditions (Synthecon and Spinner flask) relative to LKS cells purified from BM and FL. Expression levels (n = 3) were compared for genes encoding cell surface markers of hematopoiesis (**A, B**), and blood cell markers (erythropoiesis) (**C,D**), the HSC “fingerprint” genes of Chambers et al [18] (**E,F**) and critical hematopoietic transcription factors (**G,H**). The expression levels of hematopoietic markers (**A,B**) and blood cell markers (erythropoiesis) (**C,D**) were primarily down-regulated under ESC conditions as compared to BM and FL. When compared to BM and FL, the majority of the HSC fingerprint (**E,F**) and HSC transcription factor gene expression (**G,H**) was also down-regulated. (*) represent gene- expression values of statistical significance (p<0.05).

To evaluate hematopoiesis, we compared fold changes in gene expression levels of hematopoietic cell-surface markers and hematopoiesis-related molecules under each *in vitro* condition to BM and FL (**Figure 4**). In general, we observed a slight down-regulation of hematopoietic markers in ESC-derived cells compared to BM- and FL-derived LKS cells, with the exception of Sca-1 up-regulation in all three conditions. Specifically, Synthecon™ cultures showed high levels of Sca-1, consistent with our previous results [14].

The expression of additional cell-surface markers (CD150, Thy-1, CD48 and CD41) implicated in heterogeneity of murine LKS cells [30] was primarily down-regulated **(** **Figures 4A,B** **)**. However, only the expression of CD150 and CD41 on Synthecon and Spinner flask conditions compared to BM was statistically significantly reduced. Additionally, the expression levels of blood cell markers were mostly down-regulated in ESC conditions compared to BM and FL. Expression of embryonic hemoglobins, Hbb-bh1 and Hbb-y, was significantly higher in Static than Synthecon or Spinner flasks **(** **Figures 4C,D** **)**. The expression of mature hematopoietic cell marker, CD45, was statistically significantly down-regulated in all culture conditions compared to BM **(** **Figure 4A** **)** and in Synthecon and Spinner flask conditions compared to FL (**Figure 4B**).

We next determined whether we could detect a “HSC fingerprint” as described by Chambers et al. [18]. The HSC fingerprint genes analyzed **(** **Figures 4E,F** **)** were significantly more down-regulated in Synthecon or Spinner flask conditions than Static when compared to BM and FL. In contrast, *Tek*, an endothelial-specific receptor tyrosine kinase, was up-regulated in Static cultures relative to BM and FL (**Figures 4E,F**). Furthermore, the expression of several transcription factors critical to hematopoiesis, specifically *Scl*, *Hoxb4*, *Runx1* and *Hhex* [31]–[34], was reduced in LKS cells derived from Synthecon and Spinner conditions relative to those from Static-cultures **(** **Figures 4G,H** **)**.

### Non-Hematopoietic Lineage Gene Expression in ESC Cultures Compared to Bone Marrow and Fetal Liver

**Figure 5:**
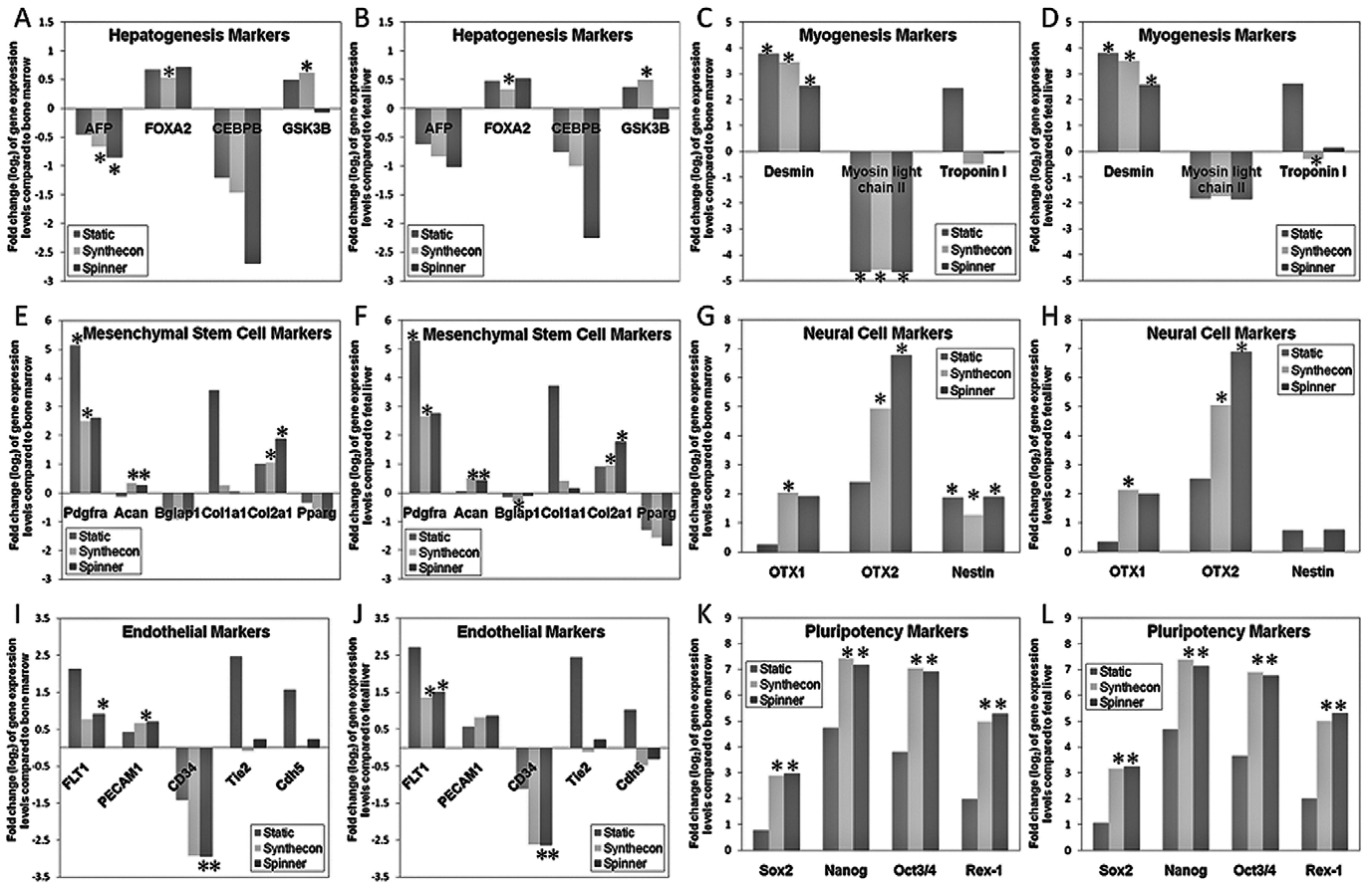
Differences in LKS cell gene expression. Fold changes of lineage-specific genes differentially regulated in Lin-ckit+Sca-1+ cells derived from Static and bioreactor culture conditions (Synthecon and Spinner flask) as compared to LKS cells derived from BM and FL. Expression levels (n = 3) were compared for genes representative of hepatogenesis (**A, B**), myogenesis (**C,D**), mesenchymal stem cells (MSC; **E,F**), neuronal cells (**G,H**), endothelial lineage (**I,J**) and transcriptional regulators of pluripotency (**K,L**). Under all ESC conditions we observed up-regulation of the hepatogenesis transcription factor, Foxa2 (**A,B**), the myogenesis intermediate filament, Desmin (**C,D**), several neuronal markers (**G,H**), and the majority of MSC markers (**E,F**). Expression of transcriptional regulators of pluripotency (**K,L**) were reduced most significantly under Static culture conditions. (*) represent gene-expression values of statistical significance (p<0.05).

We were also interested in investigating non-hematopoietic lineage-committed genes expressed in these ESC-derived Lin-ckit^+^Sca-1^+^cells. ESC conditions generated similar expression patterns for hepatogenesis and myogenesis markers **(** **Figures 5A–D** **)**. Conversely, we observed higher expression of neuronal markers in Synthecon and Spinner flasks than in Static cultures **(** **Figures 5G,H** **)**. We observed high expression of most mesenchymal stem cell (MSCs) markers in all ESC-derived Lin-ckit^+^Sca-1^+^ cells **(** **Figures 5E,F**) as well as endothelial cell markers, with the exception of CD34 **(** **Figures 5I, J** **)**.

Lastly, high expression of transcriptional regulators of ESC pluripotency, (eg, *Oct-4, Sox2, Nanog* and *Rex-1* [21], [35]) is expected as these ESC markers are not expressed in BM and FL-derived LKS cells **(** **Figures 5K,L** **)**; however, the expression was down-regulated compared to undifferentiated ESCs (data not shown). The expression levels in Static cultures were significantly lower than in Dynamic cultures.

### Hematopoietic Lineage Differentiation in ESC Cultures

**Figure 6:**
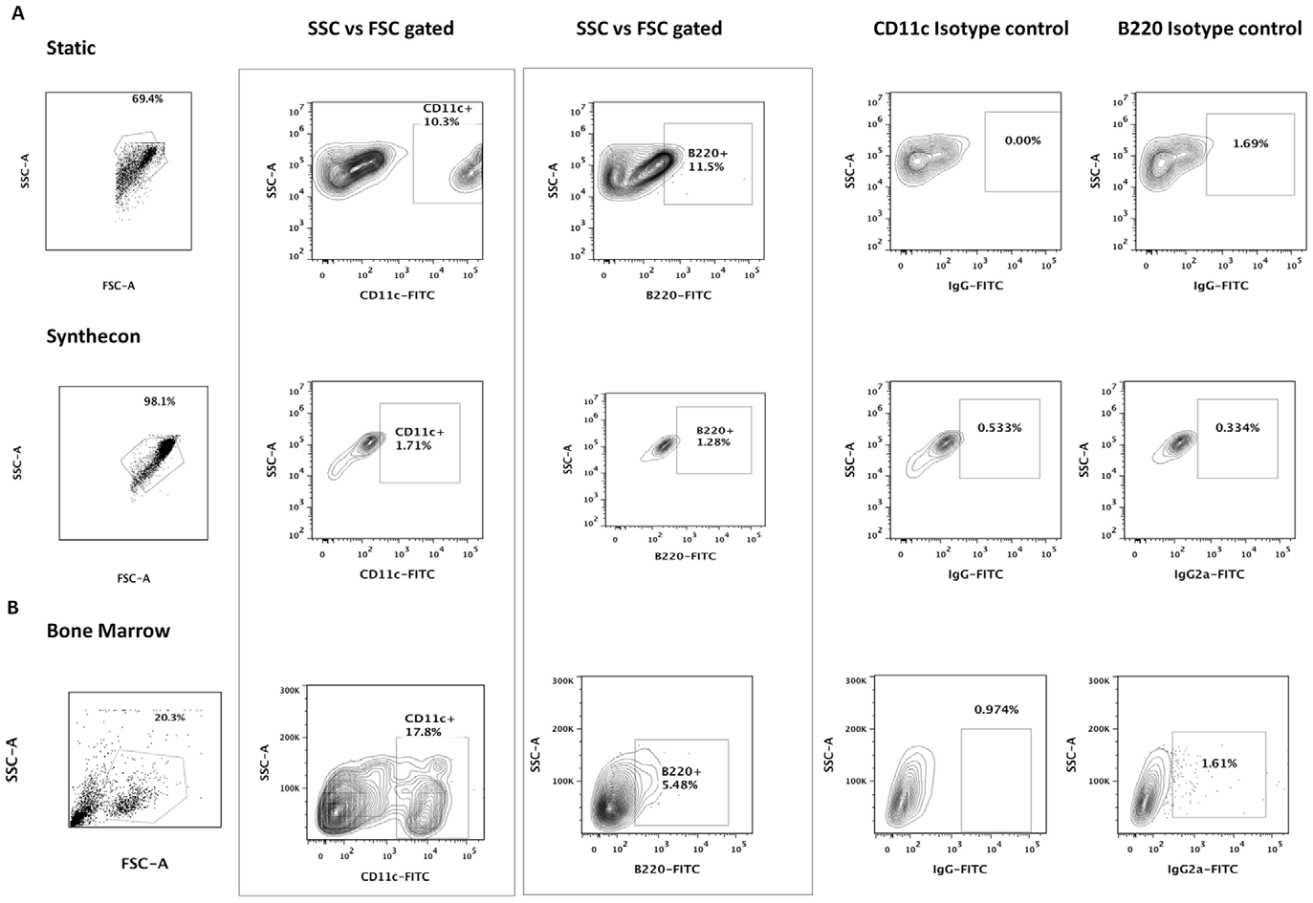
Functionality of various LKS cells. Hematopoietic differentiation of *in-vitro and in-vivo* derived Lin-ckit+Sca-1+ cells. **A)** Lin-ckit+Sca-1+ *in-vitro* derived ESCs from Static and Dynamic (Synthecon) culture conditions, as well as **B)** *in-vivo* isolated LKS cells from Bone marrow (BM) were cultured for 7 days in the presence of GM-CSF, IL-4 and IL3. Approximately 10.3% of Static-derived cells (gated on FSC-A and SSC-A) are CD11c+ (Dendritic-like cells) and about 11.5% express B220+ (B cell-like progenitors). No significant expression of CD11c or B220 in Synthecon-derived cells is observed following differentiation. As expected, Lin-ckit+Sca-1+ BM cells also differentiate into CD11c and B220+ hematopoietic progenitors.

The ability of ESC-derived progenitors to function identically to native adult stem cells is critical for their eventual clinical applicability. We evaluated whether ESC-derived LKS cells form hematopoietic colonies and compared them with BM-derived LKS cells. Unlike BM-derived cells, LKS cells derived from ESCs under either static or dynamic culture conditions, failed to form hematopoietic colonies (**see Figure S2**). Further evaluation of cells differentiated from various LKS populations demonstrated that BM and Static-derived cells were able to differentiate into B cell-like progenitors (B220+) and into dendritic-like cells (CD11c+/high) (**Figure 6**). In contrast, no significant expression of B220 and CD11c was observed in the differentiation cultures from bioreactor (Synthecon) derived-LKS cells, indicating impaired hematopoietic potential.

## Discussion

LKS cells derived from FL and BM have been well characterized in the past [23], [25], [36]. In addition several groups have reported extensive gene expression analysis of ESCs, especially with respect to their “stemness” and pluripotency [16], [17], [35]. Moreover, bioreactor cultures have shown the ability to generate hematopoietic cells from both mice [37], [38] and human [39] ESCs that are capable of forming hematopoietic colonies. Our laboratory has also shown higher colony forming ability and dendritic cell differentiation of ESC-derived hematopoietic cells in scaffold-based bioreactor cultures compared to static cultures [11]. However, whether native BM or FL-derived LKS cells are similar to those derived *in vitro* from differentiated ES or iPS cells, has not been addressed. In this work, we compared the overall gene expression profile of ESC-generated Lin-ckit+Sca-1+ cells, *in vitro*-differentiated in Static suspension cultures, dynamic Synthecon rotary-wall bioreactors and stirred tank-type (Spinner flask) bioreactors to Lin-ckit+Sca-1+ cells isolated directly from BM and FL.

Among the cell-surface markers implicated in heterogeneity of HSC population (e.g. CD150, Thy-1, CD48 and CD41), CD150 expression has been shown to be higher in *in vivo* isolated LKS cells than in the more differentiated multipotent progenitors or CD45^+^ BM cells [40]. CD41 has been reported to mark the onset of primitive and definitive hematopoiesis in the murine embryo [41]. Most yolk-sac hematopoietic progenitors express CD41, but only a minority of BM and FL hematopoietic progenitors express this antigen [42]. Interestingly in our study, CD150 and CD41 expression was statistically significantly reduced relative to BM in only Synthecon™ and Spinner flask conditions (**Figure 4A**). We suggest that the up-regulation of CD41 in static compared to FL reflects an earlier hematopoietic embryonic stage of this population, compared to FL-derived HSCs **(** **Figure 4B** **)**. The down-regulation of CD45 **(** **Figure 4A,B** **)** was expected since we isolated Lin-ckit^+^Sca-1^+^ cells at an early time-point (day 7 of ESC differentiation). It has been shown previously that CD45 is a late maturation marker of hematopoiesis whereas CD41 expression defines early steps of hematopoiesis [43]. Additionally, the up-regulation of embryonic hemoglobins, Hbb-bh1 and Hbb-y, compared to bone marrow and fetal liver **(** **Figures 4C,D** **)** confirms the earlier embryonic stage of the static population.

In addition, our results indicate that among the three different *in vitro* culture conditions, the lin-ckit+Sca-1+ cell population from Static cultures at Day 7 shows the highest similarity in gene expression compared to BM- and FL-derived HSCs. This increased similarity of Static-derived LKS cells to the cells derived from BM and FL might suggest a higher hematopoietic differentiation potential for repopulating blood cells in cellular therapies compared to cells from Spinner flask and Synthecon and needs to be validated in the future. Although the Static-derived LKS cells have increased similarity to LKS derived from BM and FL, we have previously demonstrated that the Static system produces a lower percentage of ckit+Sca-1+ compared to Spinner flasks [14]. Therefore, the number of LKS cells produced as well as the similarity to native adult stem cells needs to be addressed when considering culture conditions for obtaining clinically relevant cell numbers. In addition, all ESC-derived Lin-ckit+Sca-1+ cells were more similar to undifferentiated ESCs than were BM- or FL-derived LKS, demonstrating the importance of combining suspension culture platforms with other directed differentiation strategies, such as biomaterial-based 3D cultures [10], [11], [15], medium supplementation etc., to ensure ESC differentiation to the desired cell types.

Due to their differences in hematopoietic gene expression when compared to BM and FL-derived LKS cells, we were interested in examining other non-hematopoietic lineage committed genes expressed in these ES-derived Lin-ckit^+^Sca-1^+^ cells. In the mesoderm, ESCs give rise to mesenchymal stem cells in addition to HSCs [44]. The up-regulation of *Pdgfra* was particularly interesting as a subset of MSCs (PDGFRalpha^+^Sca-1^+^CD45^−^Ter119^−^) from adult mouse BM has been reported to differentiate into hematopoietic niche cells, osteoblasts and adipocytes, after *in vivo* transplantation [44]. We also observed high expression of endothelial cell markers, which indicated that differentiation into mesodermal cells occurred in all cultures **(** **Figures 5I,J** **)**.

Additionally, we show that hydrodynamic culture systems (Spinner flask and Synthecon) expressed higher levels of ESC pluripotency genes compared to Static cultures (**Figures 5K,L**). This could account for greater proliferation as well as differentiation potential following culture in these systems and is an issue worth further investigation. The down-regulation of these markers compared to their levels in undifferentiated ESCs could also predict a higher probability of teratomas in recipients of potential transplantation therapies. The safety of using ESC-derived therapies must be further evaluated regardless of culture methods used for differentiation.

Finally, we show that although the various *in vitro* culture conditions generate a significant population of LKS cells, there are significant differences in the functionality of ESC-derived LKS cells between Static and Dynamic culture conditions as well as when compared to LKS cells isolated from *in vivo* hematopoietic niches (**Figure 6**). As expected, LKS population from BM cells formed hematopoietic colonies in a CFU assay and were able to differentiate into DC-like and B cell-like progenitors. In contrast, ESC-derived LKS cells, from either static or dynamic cultures, did not form hematopoietic colonies *in vitro*. However, Static-derived cells were able to differentiate into CD11c+DC-like and B220+ B cell-like progenitors similarly to BM-derived cells. In contrast, no significant expression of B220 and CD11c was observed in Bioreactor (Synthecon)-derived cells. Collectively, these results indicate that LKS alone is not a reliable marker for hematopoietic potential of *in vitro* ESC-derived cells, even though it serves as an established marker for a BM or FL population in which the HSCs are enriched. It is possible, that spontaneous differentiation of ESCs, in the absence of specific hematopoietic growth factors, cannot generate true HSCs, even though the cells express some similar surface markers. Whether true HSCs can be generated using bioreactor cultures by adding such growth factors or through other strategies remains to be evaluated. Further understanding is clearly needed to identify the conditions to generate and isolate ESC-derived hematopoietic stem and progenitor cells to achieve a cell population functionally similar to those derived *in vivo*.

It is also possible, that there are kinetic differences in the differentiation process between Static and Synthecon-derived LKS cells. For example, by day 7, when ESC-derived LKS cells were isolated from all the *in vitro* culture conditions, the differentiation process may not have been completed in Synthecon-derived cells. This could explain the reduced differentiation efficacy in Synthecon cultures and is corroborated by a higher expression of pluripotency- associated genes in Synthecon as compared to Static. The reduced differentiation ability of bioreactors compared to Static differentiation protocols has been previously demonstrated for osteogenic and chondrogenic differentiation [45]. The kinetic effects should be further explored in future studies to conclusively determine if spontaneous ESC differentiation under static or bioreactor cultures can generate LKS cells with *in vivo*-like hematopoietic potential.

The gene expression profile of purified mouse adult HSCs has been previously studied by other groups [16]–[22]. Their goal was to compare the gene expression profiles of native adult and embryonic LKS HSC populations isolated from different tissues and under different conditions or to decipher genes specifically expressed in embryonic stem cells during pluripotency and differentiation (reviewed in [23]). To our knowledge, this is the first study to compare the gene expression profile of Lin-ckit^+^Sca-1^+^ cells derived from mouse ESCs *in vitro* under static and dynamic culture conditions to native BM and FL-derived HSCs. These results should help delineate the similarities and differences between native and ESC-derived cells, as well as identify the culture conditions to generate and isolate cell populations containing high HSC activity *in vitro*.

### Conclusion

We conclude that *in vitro* culture conditions significantly affect the transcriptome and functionality of ESC-derived LKS cells. Although all in vitro differentiated LKS cells were lineage negative and expressed ckit and Sca-1, these cells, especially those obtained from dynamic cultures, are significantly different from native LKS cells isolated from BM or FL. We have showed that although, Static culture-derived cells are able to further differentiate into myeloid and lymphoid lineages, they lack hematopoietic colony forming ability in clonogenic assays. Interestingly, Lin-ckit+Sca-1+ cells derived through spontaneous ESC differentiation in bioreactor cultures, not only are unable to form hematopoietic colonies *in vitro*, they also lack both myeloid and lymphoid differentiation potential, indicating that these cells are not committed hematopoietic progenitors, but they could represent a more stem cell-like population. Further studies, including in vivo repopulation assays should be performed in the future to definitively understand whether these cells can differentiate into blood cells *in vivo*.

## Supporting Information

Figure S1 (a) Post sort analysis of ckit+Sca-1+ lineage negative sorted cells isolated from *in-vitro* differentiated mouse ES cells under Dynamic (Synthecon) and Static cultures. Similarly, re-analysis of ckit+Sca-1+ lineage negative BM sorted cells is shown. For all conditions the ckit+Sca-1+ population was >95% pure. Similar results were obtained for Dynamic (Spinner cultures) as well as Fetal liver isolated cells (results no shown). (b) Mean Fluorescence analysis (MFI) of ckit and Sca-1 expression levels in ckit+Sca-1+ sorted cells from each condition. Although all conditions expressed both ckit and Sca1, differences were seen in the MFI between different conditions. Dashed lines, *p*<0.05 when compared to other conditions as indicated, ANOVA.
(TIFF)

Figure S2 *In-vitro* colony formation unit (CFU) assay. Types of hematopoietic colonies detected after 14 days of seeding Lin-ckit+Sca-1+ BM cells (1000cells/35 mm dish).
(TIFF)

Table S1 Enriched GO terms for up-regulated genes in ES culture conditions compared to bone marrow Lin-ckit+Sca-1+ cells.
(DOCX)

Table S2 Enriched GO terms for down-regulated genes in ES culture conditions compared to bone marrow Lin-ckit+Sca-1+ cells.
(DOCX)

Table S3 Enriched GO terms for up-regulated genes in ES culture conditions compared to fetal liver Lin-ckit+Sca-1+ cells.
(DOCX)

Table S4 Enriched GO terms for down-regulated genes in ES culture conditions compared to fetal liver Lin-ckit+Sca-1+ cells.
(DOCX)

Table S5 List of genes that are up-regulated (5-fold difference) in *in-vitro* derived Lin-ckit+Sca-1+ ES cells (Static, Dynamic (Spinner+Synthecon)) compared to native Lin-ckit+Sca-1+ cells (BM+FL).
(DOCX)

Table S6 List of genes that are down-regulated (5-fold difference) in *in-vitro* derived Lin-ckit+Sca-1+ ES cells (Static, Dynamic (Spinner+Synthecon)) compared to native Lin-ckit+Sca-1+ cells (BM+FL).
(DOCX)
