# Supplementary figures and images for: Gene Expression Profile and Functionality of ESC-Derived Lin-ckit+Sca-1+ Cells Are Distinct from Lin-ckit+Sca-1+ Cells Isolated from Fetal Liver or Bone Marrow
Source: PLoS One. 2012 Dec 27;7(12):e51944. doi: 10.1371/journal.pone.0051944 (PMC3531429; doi:10.1371/journal.pone.0051944)

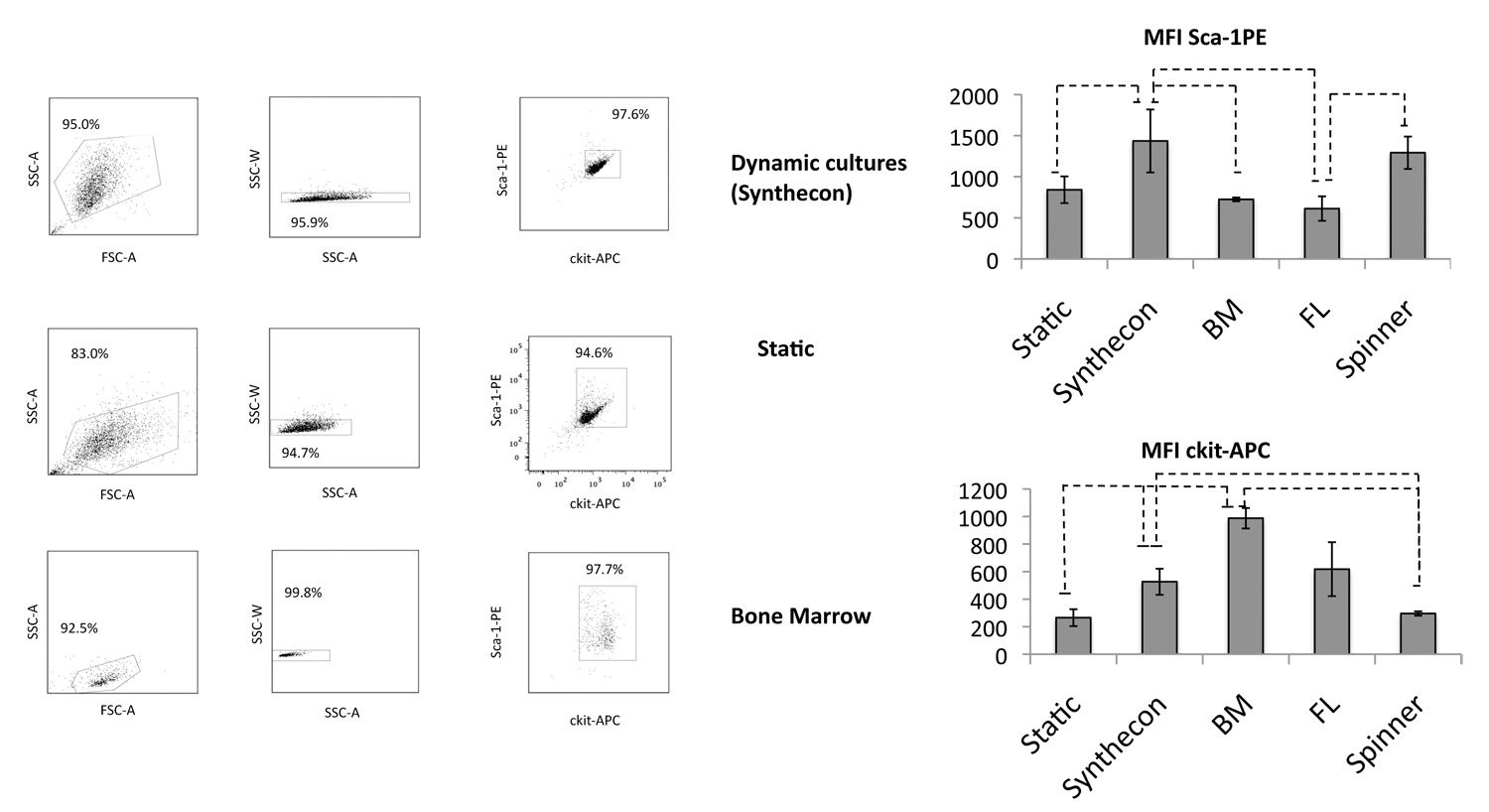

Supplement: Figure S1 — (a) Post sort analysis of ckit+Sca-1+ lineage negative sorted cells isolated from in-vitro differentiated mouse ES cells under Dynamic (Synthecon) and Static cultures. Similarly, re-analysis of ckit+Sca-1+ lineage negative BM sorted cells is shown. For all conditions the ckit+Sca-1+ population was >95% pure. Similar results were obtained for Dynamic (Spinner cultures) as well as Fetal liver isolated cells (results no shown). (b) Mean Fluorescence analysis (MFI) of ckit and Sca-1 expression levels in ckit+Sca-1+ sorted cells from each condition. Although all conditions expressed both ckit and Sca1, differences were seen in the MFI between different conditions. Dashed lines, p<0.05 when compared to other conditions as indicated, ANOVA. (TIFF) [file pone.0051944.s001.tiff]

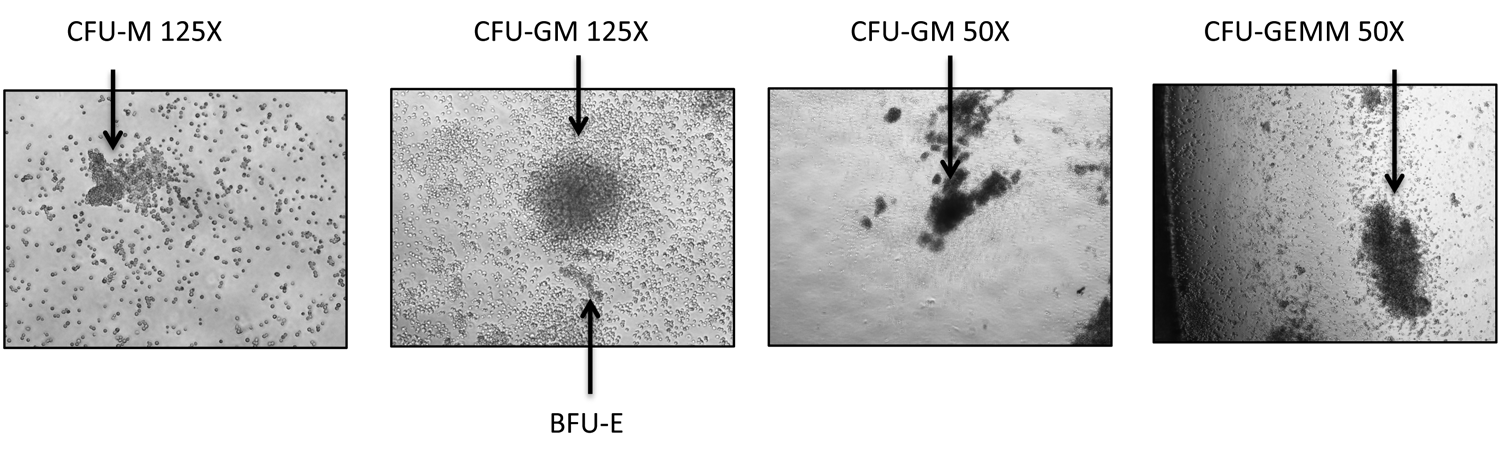

Supplement: Figure S2 — In-vitro colony formation unit (CFU) assay. Types of hematopoietic colonies detected after 14 days of seeding Lin-ckit+Sca-1+ BM cells (1000cells/35 mm dish). (TIFF) [file pone.0051944.s002.tiff]
