# Supplementary material for: Gene Expression Profile and Functionality of ESC-Derived Lin-ckit+Sca-1+ Cells Are Distinct from Lin-ckit+Sca-1+ Cells Isolated from Fetal Liver or Bone Marrow
Source: PLoS One. 2012 Dec 27;7(12):e51944. doi: 10.1371/journal.pone.0051944 (PMC3531429; doi:10.1371/journal.pone.0051944)
Supplement: Table S1 — Enriched GO terms for up-regulated genes in ES culture conditions compared to bone marrow Lin-ckit+Sca-1+ cells. (DOCX) [file pone.0051944.s003.docx]

| Culture Condition | GO term | GO ID | Ontology | Number of genes | P value |
| --- | --- | --- | --- | --- | --- |
| *all ES cells* |  |  |  |  |  |
|  | basement membrane | GO:0005604 | CC | 8 | 3.73E-06 |
|  | anatomical structure development | GO:0048856 | BP | 27 | 3.81E-05 |
|  | multicellular organismal development | GO:0007275 | BP | 28 | 8.61E-05 |
|  | protein homodimerization activity | GO:0042803 | MF | 6 | 0.00216 |
|  | cell leading edge | GO:0031252 | CC | 5 | 0.00607 |
|  | biological adhesion | GO:0022610 | BP | 11 | 0.00607 |
|  | calcium ion binding | GO:0005509 | MF | 11 | 0.0455 |
|  | plasma membrane | GO:0005886 | CC | 17 | 0.0467 |
| *static only* |  |  |  |  |  |
|  | multicellular organismal development | GO:0007275 | BP | 20 | 0.000162 |
|  | anatomical structure development | GO:0048856 | BP | 18 | 0.000498 |
|  | ion binding | GO:0043167 | MF | 2 | 0.0792 |
|  | insulin-like growth factor binding | GO:0005520 | MF | 2 | 0.0792 |
|  | fatty-acyl-CoA synthase activity | GO:0004321 | MF | 1 | 0.0792 |
| *dynamic only* |  |  |  |  |  |
|  | cell junction | GO:0030054 | CC | 17 | 0.000831 |
|  | organ morphogenesis | GO:0009887 | BP | 20 | 0.0318 |
|  | lipid binding | GO:0008289 | MF | 15 | 0.032 |
|  | stem cell differentiation | GO:0048863 | BP | 3 | 0.0559 |
|  | positive regulation of osteoblast differentiation | GO:0045669 | BP | 3 | 0.0559 |
|  | embryo development | GO:0009790 | BP | 17 | 0.0854 |

Subset of GO groups with the lowest p-values from unique term lineages.
