# Supplementary material for: Gene Expression Profile and Functionality of ESC-Derived Lin-ckit+Sca-1+ Cells Are Distinct from Lin-ckit+Sca-1+ Cells Isolated from Fetal Liver or Bone Marrow
Source: PLoS One. 2012 Dec 27;7(12):e51944. doi: 10.1371/journal.pone.0051944 (PMC3531429; doi:10.1371/journal.pone.0051944)
Supplement: Table S2 — Enriched GO terms for down-regulated genes in ES culture conditions compared to bone marrow Lin-ckit+Sca-1+ cells. (DOCX) [file pone.0051944.s004.docx]

| Culture Condition | GO term | GO ID | Ontology | Number of genes | P value |
| --- | --- | --- | --- | --- | --- |
| *all ES cells* |  |  |  |  |  |
|  | antigen processing and presentation | GO:0019882 | BP | 8 | 5.49E-07 |
|  | MHC protein complex | GO:0042611 | CC | 6 | 5.49E-07 |
|  | plasma membrane | GO:0005886 | CC | 23 | 1.19E-05 |
|  | water channel activity | GO:0015250 | MF | 2 | 0.0224 |
|  | defense response | GO:0006952 | BP | 10 | 0.000751 |
|  | fluid transport | GO:0042044 | BP | 3 | 0.000944 |
| *static only* |  |  |  |  |  |
|  | nucleosome | GO:0000786 | CC | 4 | 2.47E-06 |
|  | cellular component organization | GO:0016043 | BP | 5 | 0.00326 |
| *dynamic only* |  |  |  |  |  |
|  | immune system process | GO:0002376 | BP | 42 | 4.49E-19 |
|  | hematopoiesis | GO:0030097 | BP | 18 | 1.42E-05 |
|  | immune system development | GO:0002520 | BP | 18 | 0.000127 |
|  | cell activation | GO:0001775 | BP | 14 | 0.00214 |
|  | regulation of phagocytosis | GO:0050764 | BP | 4 | 0.00313 |
|  | chemokine receptor binding | GO:0042379 | MF | 5 | 0.00752 |
|  | defense response | GO:0006952 | BP | 26 | 1.88E-16 |
|  | multi-organism process | GO:0051704 | BP | 8 | 0.0466 |
|  | response to external stimulus | GO:0009605 | BP | 26 | 8.59E-13 |
|  | phagocytosis | GO:0006909 | BP | 8 | 3.30E-05 |
|  | external side of plasma membrane | GO:0009897 | CC | 10 | 0.00193 |
|  | response to chemical stimulus | GO:0042221 | BP | 16 | 0.00214 |
|  | extracellular space | GO:0005615 | CC | 48 | 0.00214 |

Subset of GO groups with the lowest p-values from unique term lineages.
