# Supplementary material for: Gene Expression Profile and Functionality of ESC-Derived Lin-ckit+Sca-1+ Cells Are Distinct from Lin-ckit+Sca-1+ Cells Isolated from Fetal Liver or Bone Marrow
Source: PLoS One. 2012 Dec 27;7(12):e51944. doi: 10.1371/journal.pone.0051944 (PMC3531429; doi:10.1371/journal.pone.0051944)
Supplement: Table S3 — Enriched GO terms for up-regulated genes in ES culture conditions compared to fetal liver Lin-ckit+Sca-1+ cells. (DOCX) [file pone.0051944.s005.docx]

| Culture Condition | GO term | GO ID | Ontology | Number of genes | P value |
| --- | --- | --- | --- | --- | --- |
| *all ES cells* |  |  |  |  |  |
|  | basement membrane | GO:0005604 | CC | 5 | 1.32E-03 |
|  | cell leading edge | GO:0031252 | CC | 5 | 1.92E-03 |
|  | anatomical structure development | GO:0048856 | BP | 16 | 3.95E-03 |
|  | cellular component movement | GO:0006928 | BP | 6 | 0.0245 |
|  | localization of cell | GO:0051674 | BP | 6 | 0.0245 |
|  | cell adhesion | GO:0007155 | BP | 7 | 0.0285 |
|  | plasma membrane | GO:0005886 | CC | 11 | 0.061 |
| *static only* |  |  |  |  |  |
|  | organ morphogenesis | GO:0009887 | BP | 7 | 0.0119 |
|  | transcription factor complex | GO:0005667 | CC | 6 | 0.0119 |
|  | organelle lumen | GO:0043233 | CC | 7 | 0.0223 |
|  | nuclear part | GO:0044428 | CC | 7 | 0.0223 |
|  | fatty-acyl-CoA synthase activity | GO:0004321 | MF | 1 | 0.0573 |
|  | hyaluronan synthase activity | GO:0050501 | MF | 1 | 0.0573 |
|  | heparin binding | GO:0008201 | MF | 2 | 0.0885 |
| *dynamic only* |  |  |  |  |  |
|  | organ morphogenesis | GO:0009887 | BP | 19 | 0.00924 |
|  | specification of symmetry | GO:0009799 | BP | 4 | 0.0641 |
|  | cell-cell junction | GO:0005911 | CC | 10 | 0.00924 |
|  | positive regulation of cellular metabolic process | GO:0031325 | BP | 15 | 0.00924 |
|  | positive regulation of osteoblast differentiation | GO:0045669 | BP | 3 | 0.0218 |
|  | sequence-specific DNA binding transcription factor activity | GO:0003700 | MF | 24 | 0.0116 |
|  | heart development | GO:0007507 | BP | 11 | 0.0156 |
|  | stem cell differentiation | GO:0048863 | BP | 3 | 0.0218 |
|  | transmembrane receptor protein serine/threonine kinase signaling pathway | GO:0007178 | BP | 7 | 0.0228 |
|  | embryo development | GO:0009790 | BP | 16 | 0.0228 |
|  | basement membrane | GO:0005604 | CC | 6 | 0.045 |
|  | regulation of meiosis | GO:0040020 | BP | 2 | 0.0911 |

Subset of GO groups with the lowest p-values from unique term lineages.
