# Supplementary material for: Gene Expression Profile and Functionality of ESC-Derived Lin-ckit+Sca-1+ Cells Are Distinct from Lin-ckit+Sca-1+ Cells Isolated from Fetal Liver or Bone Marrow
Source: PLoS One. 2012 Dec 27;7(12):e51944. doi: 10.1371/journal.pone.0051944 (PMC3531429; doi:10.1371/journal.pone.0051944)
Supplement: Table S4 — Enriched GO terms for down-regulated genes in ES culture conditions compared to fetal liver Lin-ckit+Sca-1+ cells. (DOCX) [file pone.0051944.s006.docx]

| Culture Condition | GO term | GO ID | Ontology | Number of genes | P value |
| --- | --- | --- | --- | --- | --- |
| *all ES cells* |  |  |  |  |  |
|  | extracellular space | GO:0005615 | CC | 12 | 2.36E-03 |
|  | defense response to bacterium | GO:0042742 | BP | 3 | 2.96E-03 |
|  | response to external stimulus | GO:0009605 | BP | 5 | 0.0218 |
|  | hydrolase activity | GO:0016787 | MF | 8 | 0.0738 |
| *dynamic only* |  |  |  |  |  |
|  | immune system process | GO:0002376 | BP | 29 | 1.42E-12 |
|  | defense response | GO:0006952 | BP | 17 | 7.14E-05 |
|  | signal transduction | GO:0007165 | BP | 47 | 0.00444 |
|  | cell communication | GO:0007154 | BP | 49 | 0.0055 |
|  | response to external stimulus | GO:0009605 | BP | 15 | 0.00444 |
|  | extracellular space | GO:0005615 | CC | 36 | 5.50E-03 |
|  | protein tyrosine phosphatase activity | GO:0004725 | MF | 6 | 0.02 |
|  | cytokine receptor activity | GO:0004896 | MF | 5 | 2.53E-02 |
|  | plasma membrane | GO:0005886 | CC | 29 | 0.0378 |

Subset of GO groups with the lowest p-values from unique term lineages. Static ESC culture only showed down-regulation of one gene within the parameters specified (differentially expressed with p<0.05 and a 5 fold difference) and therefore did not have any associated GO terms.
