# Supplementary material for: Gene Expression Profile and Functionality of ESC-Derived Lin-ckit+Sca-1+ Cells Are Distinct from Lin-ckit+Sca-1+ Cells Isolated from Fetal Liver or Bone Marrow
Source: PLoS One. 2012 Dec 27;7(12):e51944. doi: 10.1371/journal.pone.0051944 (PMC3531429; doi:10.1371/journal.pone.0051944)
Supplement: Table S5 — List of genes that are up-regulated (5-fold difference) in in-vitro derived Lin-ckit+Sca-1+ ES cells (Static, Dynamic (Spinner+Synthecon)) compared to native Lin-ckit+Sca-1+ cells (BM+FL). (DOCX) [file pone.0051944.s007.docx]

| Zfp42 | Utf1 | Tcl1 | Cpsf4l | 2410116G06Rik | Gstp2 | 1700012H05Rik | Otx2 | Dnmt3l | Lrrc34 |
| --- | --- | --- | --- | --- | --- | --- | --- | --- | --- |
| Dnmt3l | Rhox9 | Slc7a3 | Pou5f1 | Aard | Echdc2 | Trh | Tcfap2c | Zic3 | Cldn4 |
| Tdh | Fbxo15 | Upp1 | Pou5f1 | Grb7 | Wfdc2 | 2410146L05Rik | Rbpms2 | Esrrb | Igfbp2 |
| Pdgfa | Tcfap2c | Gldc | Hspb1 | Fabp3 | Nanog | Cldn6 | Tubb2b | Nid2 | Punc |
| Wasf1 | Igfbp2 | Gp38 | Cdkn1c | Flrt3 | Mmp2 | Pdgfra | Ctgf | LOC100048721 | Ptn |
| Loxl1 | Sall4 | Col4a1 | Bmp4 | Rprm | Meis2 | D0H4S114 | Rhox5 | Gpc3 | Spink3 |
| Smarca1 | Fxyd6 | Acta2 | Krt8 | Pmp22 | Col18a1 | Scarf2 | Fermt2 | Gpc3 | Mest |
| Hba-x | Sparc | Pitx2 | Dlk1 | Hbb-bh1 | Capn6 | Igfbp5 | Gata6 | Dppa5 | Acta2 |
