# Supplementary material for: Gene Expression Profile and Functionality of ESC-Derived Lin-ckit+Sca-1+ Cells Are Distinct from Lin-ckit+Sca-1+ Cells Isolated from Fetal Liver or Bone Marrow
Source: PLoS One. 2012 Dec 27;7(12):e51944. doi: 10.1371/journal.pone.0051944 (PMC3531429; doi:10.1371/journal.pone.0051944)
Supplement: Table S6 — List of genes that are down-regulated (5-fold difference) in in-vitro derived Lin-ckit+Sca-1+ ES cells (Static, Dynamic (Spinner+Synthecon)) compared to native Lin-ckit+Sca-1+ cells (BM+FL). (DOCX) [file pone.0051944.s008.docx]

| S100a8 | Ctsg | S100a9 | Ela2 | Prg2 | Lcn2 | Camp | Ngp | Ms4a3 | Ear2 |
| --- | --- | --- | --- | --- | --- | --- | --- | --- | --- |
| Ear4 | Prtn3 | Fcnb | Aqp1 | Ly6c1 | Chi3l3 | Hbb-b2 | Ear6 | Cpa3 | Fcnb |
| Chi3l3 | Aqp1 | Cd27 | Lyzs | Cpa3 | Hbb-b1 | Mpo | Lyz2 | Ramp1 | Ctse |
| Mpo | Eraf | Abcb4 | Lyz | Cd69 | Hdc | C3 | Chi3l1 | Hp | EG433016 |
| Ltf | Hp | Ifitm6 | Sell | Stfa1 | Casp1 | Mpo | Serpina3g | Pglyrp1 | Hp |
| Klf1 | Rac2 | Kel | Plac8 | Trim10 | Cd53 | Coro1a | Rgs1 | Slc4a1 | Coro1a |
| Ncf4 | Coro1a | Coro1a | Btk | Arhgdib | Cd52 | Pscdbp | Gypa | F13a1 | Samsn1 |
| Was | Slc38a5 | Nfe2 | Gypc | Ccl4 | Lmo2 | Alas2 | Cd93 | Tyrobp | Sla |
| Hba-a1 | Slpi | ENSMUSG00000043795 | Ccl9 | Alox5ap | Cxcl4 | Klhl6 | Klhl6 | Ccl3 |  |
